# When Error Learning Orientation Leads to Learning From Project Failure: The Moderating Role of Fear of Face Loss

**DOI:** 10.3389/fpsyg.2019.01317

**Published:** 2019-06-13

**Authors:** Wenzhou Wang, Chong Yang, Bin Wang, Xiaoxuan Chen, Bingqing Wang, Wenlong Yuan

**Affiliations:** ^1^Business School, Beijing Normal University, Beijing, China; ^2^Faculty of Business and Law, Future of Work Institute, Curtin University, Perth, WA, Australia; ^3^North China University of Technology, Beijing, China; ^4^Asper School of Business, University of Manitoba, Winnipeg, MB, Canada

**Keywords:** learning from failure, error learning orientation, positive grieving, fear of face loss, project failure

## Abstract

As the importance of failure is widely recognized, there is increasing research interest in the antecedents of learning from failure. Basing on affective event theory, the current study cast light on individuals’ cognition of error and proposed that employees with higher levels of error learning orientation tend to show more positive grieving after project failure, which in turn increases their learning from failure. Using a sample of 752 employees from 140 project teams, we found empirical evidence to support this theoretical model. Our results indicated that positive grieving mediated the relationship between error learning orientation and learning from failure. Besides, the relationship between error learning orientation and positive grieving is more positive for employees with lower levels of fear of face loss. Our findings help enrich the antecedents of learning from failure by shedding light on how and when error learning orientation matters. Theoretical and practical implications are discussed.

## Introduction

Although failure typically hinders achieving organizational goals, it also provides a unique and valuable opportunity for individuals and organizations to learn (Josefy et al., 2017; Dahlin et al., 2018). Previous studies have shown that learning from failure is conducive to organizational and individual performance (e.g., increased financial performance and lower accident rates, Hirak et al., 2012; Madsen, 2009). Given the important role of learning from failure, various studies have started to explore its antecedents. Fruitful studies have explored the crucial roles of organizational/team level factors in influencing learning behavior after failure such as organizational culture (Ashforth and Kreiner, 2002), leadership (Ye et al., in press), and team psychological safety climate (Edmondson, 1999). At individual level, previous studies usually focus on personal resources that can help to cope with failure such as coping orientation, emotional stability, and failure experience (Shepherd et al., 2011; Zhao, 2011), while little is known regarding the role of individuals’ cognitions of errors or failures.

From the theoretical view, cognitive process occurs earlier than emotional and behavioral responses after failure (Zhao and Olivera, 2006). According to the affective events theory (AET; Weiss and Cropanzano, 1996), after negative work events, such as project failure, individuals will first evaluate this event and show specific emotions and behaviors. From the practical view, personal resources (e.g., failure experiences) indicates the potential resources or capability that employees can invest to cope with failure. However, they may exert less efforts to learn from failure if they cannot realize the values of the failed events. Thus, individuals’ cognitions of errors or failures are theoretically and practically important.

Studies building on AET (Weiss and Cropanzano, 1996) and Zhao and Olivera’s (2006) theory usually emphasis the cognitive reactions after failure (e.g., cognitive appraisal of the current failed event). Moving beyond their approaches, we pay attention to individuals’ relatively stable mindset of errors or failures-error learning orientation, which refers to the perception of the potential value delivered by errors (van Dyck et al., 2005). Accumulated empirical evidence has shown that individuals with higher levels of error learning orientation tend to show better performance and more innovative behaviors (e.g., Arenas et al., 2006; Bell and Kozlowski, 2008). With respect to project failure, we contend that individuals with higher levels of error learning orientation are more likely to find the bright sides (e.g., useful information for promoting performance) when they experience failure and will therefore exert more efforts to learn from failure.

To better understand the underlying mechanism between error learning orientation and learning from failure, we focus on the mediating role of grief through the theoretical prism of AET. Shepherd (2003) first incorporated grief from psychological literature into the studies on commercial failure and theorized it as a negative emotional response to the failed events. Blau (2006) identified that grief actually encompasses a set of emotions. Thus, he further divided these emotions into negative grieving (i.e., denial, anger, negotiation, and depression) and positive grieving (i.e., exploration and acceptance). Negative grieving will lead to a series of undesirable consequences, such as low cognitive performance and reduced organizational commitments (e.g., Paterson and Cary, 2002; Conway et al., 2017). In contrast, positive grieving can help individuals positively cope with a negative experience, such as more exploring behaviors (Blau, 2007). In this study, we proposed that individuals high on error learning orientation will regard failure as a good chance for development; thus, they will show more positive grieving after failure and are more willing to learn from the failed project.

Affective events theory also indicates the moderating role of personal traits in the relationship between the cognition and induced emotion (Weiss and Cropanzano, 1996). That is, such relationship will vary with individuals. Particularly in Eastern cultures, face (or social images) is closely associated with “one’s dignity, self-respect, feeling of social concern, and ability to fill social obligations in front of other people” (Bedford and Hwang, 2003, p. 136); therefore, individuals exhibit fear of face loss. As a project is related to the reputation and competence of employees, suffering from failure will directly hurt their social image (i.e., losing face). Individuals who fear face loss tend to pay more attention to the damages to their social image after a project failure. Thus, although they may realize the potential values of failure, they will show less positive grieving. In contrast, individuals with lower levels of fear of face loss can cope with the failure more rationally and positively (i.e., show more positive grieving).

**FIGURE 1:**
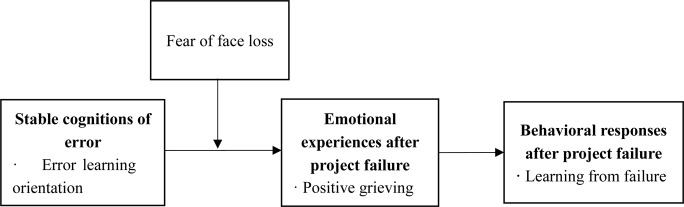
Theoretical model.

Altogether, based on AET, the current study aims to explore how error learning orientation leads to learning from failure via the induction of positive grieving after project failure and concerns the moderator of fear of face loss in the process (we summarized the theoretical model in Figure 1). By doing so, we contribute to the literature of learning from failure in three ways. First, although the theoretical work has addressed the role of cognitive factors in learning from failure (e.g., Weiss and Cropanzano, 1996; Zhao and Olivera, 2006), limited empirical studies have examined the impacts of individuals’ stable mindset or cognition of the potential value delivered by failure (i.e., error learning orientation). Thus, our study enriches the antecedents of learning from failure. Second, we employ the AET to link error learning orientation and learning from failure with positive grieving. The dominated views in learning from failure areas indicate that negative emotions are unavoidable after failure and grief impedes subsequent learning behavior (e.g., Shepherd, 2003; Zhao, 2011). However, people high on error learning orientation are more likely to find the bright sides and “positively grieve.” Our study goes beyond Shepherd’s (2003) negative theorization of grief in failure by examining positive grieving. Finally, we probed deeper understandings of personal traits (i.e., fear of face loss) in the relationship between error learning orientation and emotional response after failure. One recent qualitative study indicated the concern of face loss will reduce approach behavior facing the potential failure (Chua and Bedford, 2016). Our study further examines these personal traits after project failure in Chinese context, providing development beyond their study.

## Hypothesis Development

### Error Learning Orientation and Positive Grieving

According to AET, individuals will first evaluate the work event and show a specific affective response (Weiss and Cropanzano, 1996; Zhao and Olivera, 2006). For example, Bohns and Flynn (2013) proposed that employees will feel guilty when they believe their errors/mistakes exert negative impacts on other individuals, whereas they will show shame when these events hurt their social image. Such cognitive appraisal after events is influenced by individuals’ long-term or stable cognition. In the project failure context, error learning orientation that reelects individuals’ perception of the potential values delivered by errors (van Dyck et al., 2005), will influence how they evaluate this failed event when they experience failure, which in turn, predicts subsequent emotional and behavioral responses.

First, individuals with higher levels of error learning orientation are more likely to realize the potential values of errors, and they will, therefore, hold more positive cognition of project failures in professional life. For example, they will regard the failure as an opportunity rather than a stigma experience or symbol of incompetence (Mathieu et al., 2005). Thus, instead of blindly indulging in negative emotions after failure (Ashforth and Kreiner, 2002), they will accept the failure more easily and exert efforts to adjust their status or explore other potential solutions (Shepherd and Cardon, 2009). In contrast, for individuals with negative cognition of errors or failures, failure is more likely to be evaluated as a hindrance for their work. As a result, they will show a series of negative reactions, such as denying the failed fact (Blau, 2006, 2007).

In addition, employees high on error learning orientation will own more psychological capital, such as self-efficacy, after project failure (Jenkins et al., 2014), as they believe that past failure is a challenge in their professional career that can be solved through efforts. Thus, they will feel more confident to cope with the project failure and are willing to spend time and energy attempting to explore the causes and solutions of the failure (Jenkins et al., 2014). In contrast, individuals with lower levels of error learning orientation tend to be more vulnerable. For example, after failure, they may feel that they are not qualified for the job (Amundson, 1994). Thus, they are more likely to immerse in negative grieving. Hence, we propose that:

Hypothesis 1: Error learning orientation is positively related to positive grieving.

### Positive Grieving and Learning From Failure

Affective events theory proposes that emotions induced by specific events will influence individuals’ subsequent behaviors (Weiss and Cropanzano, 1996). For the project failure condition, emotion after project failure is regarded as one of the most important factors that hinder or enhance learning from failure (Zhao and Olivera, 2006; Shepherd and Cardon, 2009). Although most empirical results have indicated the detrimental impacts of negative emotions on learning failure, scholars have started to pay attention to the desirable consequences of negative emotions, such as guilt (Bohns and Flynn, 2013). Positive grieving is the bright side of grief (a negative emotional response; Shepherd, 2003) after negative events, and it will contribute to acceptance and exploration, thereby helping employees to learn from previous project failure (Blau, 2006, 2007, 2008).

According to Blau (2007), positive grieving manifests as two aspects: acceptance (accept the fact of failure) and exploration (eager to explore for hopeful opportunity and new possibilities). First, accepting the fact of failure will help individuals to shift their attention from immersing in negative events to reflecting this event (Ellard et al., 2017). As a result, individuals can gradually recognize the reality of failure and are not afraid of the failure or escape from it (Blau, 2007). Moreover, once individuals are immersed in the previous failure, they will not have sufficient cognitive resources to process the information embedded in the failure and therefore cannot learn useful knowledge from it (Shepherd, 2003).

Furthermore, individuals with positive grieving will show more constructive behaviors, such as exploring the solution of the failed project and the possibility for future work. They will increase communication with other individuals (Blau, 2007). In the process of discussing failures with colleagues, employees will obtain more help and feedback regarding the failure, thereby increasing their efficiency in learning from failure. In addition, exploring the causes of failures and potential solutions can enable employees to gain more useful information from the failed project (Boer et al., 2014). By summarizing the experience, employees can better understand the work events, which helps employees learn more from failure. Hence, we propose the following:

Hypothesis 2: Positive grieving is positively related to learning from failure.

In conclusion, based on AET, a positive error learning orientation enables individuals to regard failure as valuable for their work and promote positive grieving, which in turn will enhance their subsequent learning behavior (Weiss and Cropanzano, 1996). Thus, we propose that positive grieving is a mediator in the relationship between error learning orientation and members’ learning from failure:

Hypothesis 3: Positive grieving mediates the relationship between error learning orientation and learning from failure.

### The Moderating Role of Fear of Face Loss

Face plays a crucial role in eastern culture and is defined as “one’s dignity, self-respect, feeling of social concern, and ability to fill social obligations in front of other people” (Bedford and Hwang, 2003, p. 136). Losing face will make individuals feel ashamed, undermine their self-esteem (Goffman, 1955) and hurt interpersonal harmony in the group (Ho, 1991). Thus, individuals who exhibit fear of face loss are more sensitive to work events that are closely associated with their reputation and social image, and they will show more withdrawal behavior to protect their face (Zane and Yeh, 2002; Miller and Roloff, 2007). For example, fear of face loss will reduce individuals’ entrepreneurial intent (Chua and Bedford, 2016). As project failure will hurt employees’ reputation in their social life, we contend that even if individuals who fear of face loss can realize the potential values of failure, they cannot cope with these negative events with positive grieving.

First, given that face is closely related to the individual’s social status and social image (Ho, 1991), individuals who fear of face loss will pay more attention to their self-esteem and reputation. Thus, it is difficult for them to accept project failure that may cause damages to their social image. Moreover, the fear of face loss may make individuals more anxious after project failure (Zhang et al., 2011), which, in turn, affects individuals’ cognitive assessment of the failed event. For example, individuals who are afraid of losing face may think that failure is an important reason for losing face; therefore, the negative grieving induced by failure is magnified (Roseman and Evdokas, 2004), which also hinders their adjustment of their own state after failure. Thus, even if they believe that failure has potential value, it is difficult for them to accept failure.

Furthermore, face will dramatically influence individuals’ interpersonal behaviors in Eastern culture (Sue and Morishima, 1982). More specifically, employees who fear of face loss will put more resources in processing interpersonal communication to maintain and enhance face after failure (Zane and Yeh, 2002), which distracts energy for exploring the causes and solutions of the failure. Furthermore, proactive coping after failure means uncertainty and substantial risk. Therefore, to avoid losing face or restore face, individuals may behave more passively (Lemer and Keltner, 2001). Thus, even though they can realize the potential values delivered by a failed project (i.e., higher levels of error learning orientation), they would still deny the fact and own less motivations to explore (i.e., lower levels of positive grieving).

In contrast, individuals who do not fear losing face may have more confidence after project failure. Therefore, the role of error learning orientation in promoting positive grief will be enhanced:

Hypothesis 4: Fear of face loss moderates the relationship between error learning orientation and positive grieving in a way that the positive relationship will be weaker when employees are higher in the fear of face loss than lower.

## Materials and Methods

### Participants and Procedure

As the R&D teams of the high-tech industry are more vulnerable to failure in their work, we collected data from high-technology firms in Beijing that have met 60% of their annual sales from high-tech products (services) in the past one-year and at least 10% of their employees in R&D teams. We randomly selected 400 of the list of high-tech companies and then invited these companies to participate in our research. We described the purpose of the study, emphasized the confidentiality and promised to summarize the research results to each leader of the company. We also provided an endorsement letter template and asked the CEO to write an endorsement letter to encourage employees to participate in the survey. After reaching a consensus with the partner company, the company’s CEO and our research assistant identified a coordinator (typically a human resources manager); they then determined the list of teams participating in the study. We typically sent questionnaires to employees in the team’s weekly (or monthly) routine meeting. For absent participants, we left an envelope with a questionnaire and our address.

Seven hundred and seventy-four participants filled the survey. 22 reposes were excluded due to the missing data. The final sample included 752 participants from 140 teams. Approximately 50.3% of the respondents had bachelor degrees and 38.3% had master degrees. The mean respondent age was 31.70 years (range from 20 to 56 years, *SD* = 5.56), and 23% were women.

### Measures

In the first part, we measured relatively stable personal variables such as demographic information, error learning orientation, and fear of face loss. Then, we provided a short instructions and defined the project failure as “the termination of an initiative to create organizational value that has fallen short of its goals” (Shepherd et al., 2011). The participants were asked to recall their emotional experience (i.e., positive grieving) after the recent project failure and changes in their behavior since this failure (i.e. learning from failure).

#### Error Learning Orientation

We measured error learning orientation using the four-item scale adapted from van Dyck et al. (2005). The participants were instructed to indicate the extent to which they agreed with statements on a six-point scale that ranged from 1 (totally disagree) to 6 (totally agree). The items included “For me, errors are very useful for improving the work process,” “After an error, I think through how to correct it,” “After an error has occurred, I analyzed thoroughly,” and “If something went wrong, I take the time to think it through.” The reliability estimate for the scale was 0.73.

#### Fear of Face Loss

We measured fear of face loss using the five-item questionnaire adapted from Zane and Yeh (2002). The participants were instructed to indicate the extent to which they agreed with statements on a six-point scale that ranged from 1 (totally disagree) to 6 (totally agree). Sample items include “I am more affected when someone criticizes me in public than when someone criticizes me in private” and “During a discussion, I try not to ask questions because I may appear ignorant to others.” The reliability estimate for the scale was 0.60.

#### Positive Grieving

We measured positive grieving using the six-item questionnaire adapted from Blau (2007). The participants were instructed to indicate the extent to which they agreed with statements on their emotional experience after the recent project failure, with response options that ranged from 1 (totally disagree) to 6 (totally agree). Sample items include “Maybe a positive opportunity will come after the project failure” and “I will prepare for the coming change I need to make.” The reliability estimate for the scale was 0.86.

#### Learning From Failure

We measured learning from failure using the eight-item questionnaire developed by Shepherd et al. (2011). The participants were instructed to indicate the extent to which they agreed with statements on their behaviors have changed since the most recent project failure, with response options that ranged from 1 (totally disagree) to 6 (totally agree). Sample items include “I am a more forgiving person at work” and “I can more effectively run a project.” The reliability estimate for the scale was 0.91.

#### Control Variables

Except for controlling participants’ demographic variables such as age, gender, and education level, we also controlled the time since the most recent project failure at the team-level because previous studies showed that time since project failure can influence negative emotions and learning behavior (Shepherd et al., 2011).

#### Analytical Strategy

The nested structure of our data (i.e., employees are nested in project teams) raise a concern of the impacts of group/team differences. For instance, inclusive leadership is positively related to team members’ psychological safety, which in turn, further influences their learning from errors (Ye et al., in press). Similarly, organizational/team level factors such climate and leadership will also potentially influence employees’ fear of face loss, emotional and behavioral responses after project failure. Thus, though all variables in this study are all at individual level, we conducted a multilevel method to examine the hypotheses with random coefficient models to rule out the potential impacts of team-level factors. All variables at level 1 (i.e., individual level), which included error learning orientation, positive grieving, fear of face loss, and learning from failure, were group-centered to remove the between-group variance (Hofmann and Gavin, 1998). Given that we group centered the predictor and moderator before creating the interaction term, the interaction term was added into the model without centering.

Specifically, to test the effect of error learning orientation on positive grieving, we estimated a random coefficient model with HLM6, wherein positive grieving was regressed on error learning orientation. Similarly, the impact of positive grieving on learning from failure was also tested by a random coefficient. To test the moderating effect of fear of face loss, another random coefficient model was conducted, in which error learning orientation, fear of face loss, and the product term (i.e., group-centered error learning orientation × group-centered fear of face loss) were entered to predict positive grieving. Besides, we specified control variables as fixed effects in all models.

### Hypothesis Testing

We first conducted a confirmatory factor analysis (CFA) for the variables. Following Landis et al. (2000), we used the item parceling method to estimate the CFA model. As shown in Table 1, the theoretical model (i.e., the four-factor model) fits better than other alternative models, indicating the distinctiveness of our measurements. Besides, our data were collected at the same time, which may lead to the common method bias (CMB). Following Podsakoff et al. (2003), we further conducted the Harman’s single-factor test. Our results showed that no general factor accounted for a majority of the variance. The results of CFA and Harman’s single-factor test together indicate that the CMB may not be a substantial problem in this study.

**Table 1:** Comparison of measurement model.

Model	χ^2^	*df*	RMSEA	CFI	NFI
Theoretical model (four-factor model)	289.59	71	0.06	0.96	0.95
Three-factor model: error learning orientation and fear of face loss were combined into one factor	1248.18	74	0.15	0.79	0.78
Two-factor model: error learning orientation, fear of face loss, and positive grieving were combined into one factor	1804.69	76	0.17	0.70	0.69
One-factor model: all four variables were combined into one factor	2651.88	77	0.21	0.54	0.55

As shown in Table 2, error learning is positively related to positive grieving (*r* = 0.23, *p* < 0.001) and learning from failure (*r* = 0.29, *p* < 0.001), and positive grieving is positively associated with learning from failure (*r* = 0.51, *p* < 0.001), providing preliminary support for our hypothesis.

**Table 2:** Means, standard deviations, reliabilities, and correlations among study variables.

		Mean	*SD*	1	2	3	4	5	6	7
1	Gender	0.77	0.42							
2	Age	31.67	5.56	0.04						
3	Education level	4.36	0.68	0.01	0.20^∗∗∗^					
4	Error learning orientation	4.66	0.72	-0.01	0.01	0.10^∗∗∗^	(0.73)			
5	Positive grieving	4.33	0.86	-0.06	0.00	-0.06	0.23^∗∗∗^	(0.86)		
6	Fear of face loss	3.68	0.69	-0.03	-0.04	0.013	0.03	-0.017	(0.60)	
7	Learning from failure	4.59	0.85	-0.05	-0.03	-0.04	0.29^∗∗∗^	0.51^∗∗∗^	0.03	(0.91)

*N = 737–752; Cronbach’s alpha reliabilities are in parentheses on the diagonal; for gender, 1 was coded for male, and 0 was coded for female; For education level, 1 was coded for high school, 2 was coded for technical secondary school, 3 was coded for junior colleague, 4 was coded for bachelor, 5 was coded for master, and 6 was coded for PhD. ^∗∗^p < 0.01, ^∗∗∗^p < 0.001.*

As shown in Table 3, after controlling for demographic variables (i.e., gender, age, and education level), error learning orientation is significantly related to positive grieving (*b* = 0.26, *SE* = 0.05, *p* < 0.001; Model 1). Moreover, the relationship between positive grieving and learning from failure is also significant (*b* = 0.43, *SE* = 0.04, *p* < 0.001; Model 5). To further examine the indirect effect of positive grieving, we also conducted the Monte Carlo method for assessing mediation based on 20,000 simulated samples (Preacher and Selig, 2012), and our results showed that the indirect effect is significant, 95% CI [0.067 to 0.161]. Thus, Hypotheses 1–3 were supported.

**Table 3:** Multilevel estimates for models.

		Positive grieving		Learning from failure		
	Null model	Model 1	Model 2	Model 3	Model 4	Model 5
Intercept	4.58^∗∗∗^	4.50^∗∗∗^	4.48^∗∗∗^	4.79^∗∗∗^	4.85^∗∗∗^	4.73^∗∗∗^
**Level 1**						
Gender		-0.08 (0.07)	-0.08 (0.06)	-0.06 (0.06)	-0.05 (0.06)	-0.04 (0.06)
Age		-0.00 (0.01)	0.00 (0.01)	-0.00 (0.01)	-0.00 (0.01)	-0.01 (0.01)
Education level		-0.09 (0.05)	-0.09 (0.05)	-0.07 (0.05)	-0.07 (0.05)	-0.03 (0.04)
Error learning orientation		0.26^***^ (0.05)	0.26^***^ (0.05)	0.38^***^ (0.05)	0.36^***^ (0.04)	0.27^***^ (0.04)
Fear of face loss			-0.03 (0.05)		-0.01 (0.05)	-0.03 (0.05)
Error learning orientation × Fear of face loss			-0.17^*^ (0.08)		-0.06 (0.09)	-0.03 (0.08)
Positive grieving						0.43^***^ (0.04)
**Level 2**						
Time since the project failure		0.07 (0.08)	0.08 (0.08)	0.09 (0.07)	0.08 (0.07)	0.08 (0.07)
ρ^2^	0.67	0.64	0.61	0.61	0.68	0.39
*τ_00_*	0.04^∗^	0.08^∗∗∗^	0.08^∗^	0.06^∗∗^	0.06	0.09^∗∗^
ICC	0.06					

*N = 725–729; Standard error (SE) is reported in brackets; ^∗^p < 0.05, ^∗∗^p < 0.01, ^∗∗∗^p < 0.001.*

**FIGURE 2:**
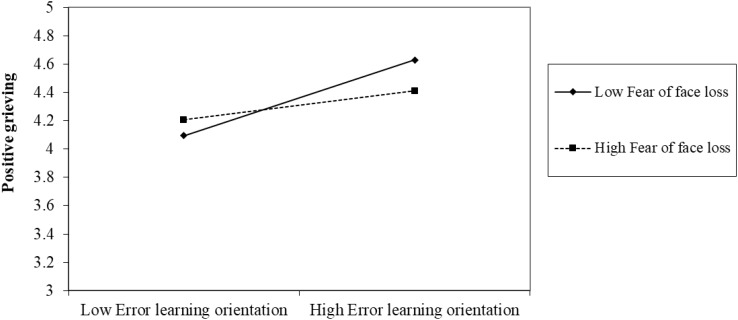
The moderating role of fear of face loss in the relationship between error learning orientation and positive grieving.

Hypothesis 4 proposed the moderating role of fear of face loss. As shown in Model 2, the interaction term (i.e., error learning orientation × fear of face loss) is significant (*b* = -0.17, *SE* = 0.08, *p* < 0.05). Following Cohen and Cohen (1983), we defined a high fear of face loss as plus one *SD* from the mean and defined a low fear of face loss as minus one *SD* from the mean. As shown in Figure 2, the relationship between error learning orientation and positive grieving is positive for the employees with lower levels of fear of face loss (simple slope = 0.38, *p* < 0.001), whereas this relationship became weaker for the employees with higher levels of fear of face loss (simple sloe = 0.14, *p* < 0.05). Thus, Hypothesis 4 was supported.

## Discussion

In the current study, we developed and examined how and when error learning orientation leads to learning from failure. Our results indicated that positive grieving mediates the relationship between error learning orientation and individuals’ learning from failure. Moreover, we found that fear of face loss moderates the impacts of error learning orientation such that the relationship between error learning orientation and positive grieving is more positive for employees with lower levels of fear of face loss.

### Theoretical Contribution

The theoretical contributions of the current study are threefold. First, we enrich the research on the antecedents of learning from failure. Though several conceptual papers pointed the crucial role of cognitive factors in predicting learning from failure (Zhao and Olivera, 2006; Shepherd and Cardon, 2009), limited empirical studies addressed their propositions/theories. Consist with their theoretical work, our results supported that positive cognition of errors or failures can contribute to more learning from failure. Moreover, most past scholars focus on the cognitive reactions after failure (Zhao and Olivera, 2006; Shepherd and Cardon, 2009), but less is known about the effects of stable mindset of error/failure (i.e., error learning orientation). Our study moved beyond their approaches and showed individuals’ stable perception of the potential value delivered by errors will predict their learning behavior when they experience failure.

Second, we explored the mediating role of positive grieving in the relationship between error learning orientation and learning from failure. Though Shepherd (2003) introduced the grief to the literature of learning from failure, there is limited evidence to further examine and develop his assumptions. Our studies revealed that individuals high on error learning orientation will specifically show more positive grieving after project failure, which in turn, enhances subsequent learning from failure. Thus, this study provided empirical evidence of grief in the failure context and revealed its bright side. Most previous studies have argued that negative emotions (e.g., grief) would suppress individuals’ learning behavior. For example, Nolen-Hoeksema et al. (1994) considered that grief induced by the failure is a hard obstacle for learning. However, recent research has started to pay attention to the bright side of negative emotions. For example, Bohns and Flynn (2013) suggest that guilt (a specific emotion caused by failure) will enhance learning after failure; however, the empirical evidence remains inadequate. Following this theoretical trend, our findings contributes to the literature by providing new insights into the role of positive grieving.

Finally, our study also captures the moderating role of individuals’ personal factors-fear of face loss. According to AET, the relationship between cognition and emotion will vary with individuals (Weiss and Cropanzano, 1996). Our results indicated that personal factors, such as fear of face loss, will weaken the relationship between error learning orientation and positive grieving after failure. In fact, various boundary condition variables in learning from failure have been examined in previous studies. However, scholars have mainly paid attention to self-regulation behaviors (e.g., emotion regulation, Fang He et al., 2018), emotion-related personal traits (e.g., emotion stability, Zhao, 2011), and contextual variables (e.g., error management culture, van Dyck et al., 2005). Following the recent call for more attention on self-conscious emotions (e.g., guilt, shame, and embarrassment) after a failed event (Bohns and Flynn, 2013), scholars have started to explore complex emotional responses embedded in interpersonal relationships. Thus, individuals’ behaviors or emotions after failure will be influenced by other individuals’ real or perceived reactions. In the current study, employees low on fear of face loss do not care about other individuals’ negative reactions to their failure, whereas individuals high on fear of face loss are more sensitive to other individuals’ (both real or perceived) reactions. Although the term “face” was developed from eastern culture, the concerns regarding social image generally exist in other cultures. Thus, our study provides new directions for future studies to explore the boundary conditions in the process of learning from failure.

### Practical Contributions

Our research also provides several practical contributions. For team leaders and mangers, they should conduct effective error management. For example, it’s necessary to build the learning climate (Cortini et al., 2016) and error management culture (van Dyck et al., 2005), which will be conducive for employees to learn from failure. By doing this, employees will have a more positive understanding of the value of failure (i.e., error learning orientation), thereby enhancing learning behavior after failure. Besides, error management can also help to increase the tolerance of failure. Hence, employees will concern less about the face loss and can behave more positively toward to failure.

For individuals, they should build a holistic understanding of project failure. Given that failure is frequent and common in the current commercial world, it’s important to glean knowledge from previous experience and make changes. Though it’s difficult for people to control the raised negative emotions after failed events, they can grieve in a positive way.

### Limitations and Future Research

In addition to the previously described contributions, our study also has several limitations. First, the data employed were only collected from R&D teams in China, which restricts the generalizability of our conclusions. Besides, the unique sample and voluntary participation may also influence the distributive qualities of the dataset. Thus, future investigations could examine this theoretical model with samples from different industries using random sampling method. Moreover, the scales employed originated from international maturity scales, which may not be fully adapted in China contexts. For example, individuals in different cultures have different attitudes on “face.” Compared with western culture, individuals in eastern culture may pay more attention to protect their “face” (Zane and Yeh, 2002). In view of this difference, it is necessary to try to conduct cross-cultural research in the future to increase the richness and universality of our results.

Second, our data were collected from the single time point and single source (i.e., self-report approach), which may lead to the CMB. Though we conducted the Harman’s one-factor analysis and CFA to test the risk of CMB in the current study, multiple sources of raters are recommended for future research to reduce the influence of CMB. For example, coworkers or leaders can report the changes in behavior of the focal employee. Besides, time-lagged or repeated measures will also help to reduce CMB. By doing this, scholars can trace employees’ changes after project failure and probe deeper into the underlying emotional process.

Third, the current study only employs the quantitative method to capture employees’ emotional and behavioral responses after project. In fact, recalling the failure experience may contribute to biases because participants are easily influenced by their emotion states or social desirability. Accordingly, mixed methods are recommend for future studies. For example, scholars can employ qualitative approaches, thereby probing deeper into the underlying mechanisms and addressing the complex cognition-emotion-behavior relationship.

## Conclusion

The current study aims to answer how and when error learning orientation contributes to learning from failure after project failure. Our findings indicated that positive grieving after project failure acts as the mediating role between error learning orientation and learning from failure, and the fear of face loss will weaken the positive impacts of error learning on positive grieving. Thus, we enriched the research regarding the antecedents of learning from failure and added new insights into positive grieving in the workplace.

## Ethics Statement

All procedures performed in studies involving human participants were in accordance with the ethical standards of the institutional and/or national research committee and with the 1964 Helsinki declaration and its later amendments or comparable ethical standards with written informed consent from all subjects. This study was carried out in accordance with the Declaration of Helsinki and ethical guidelines and approved by the Human Research Ethics Committee (HREC) at the Beijing Normal University.

## Author Contributions

WW, CY, and BW substantially contributed to the conception and the design of the work as well as in the analysis and interpretation of the data. CY and XC prepared the draft. BQW and WY reviewed and revised it critically and gave important intellectual input.

## Conflict of Interest Statement

The authors declare that the research was conducted in the absence of any commercial or financial relationships that could be construed as a potential conflict of interest.
